# Precision Medicine for Lysosomal Disorders

**DOI:** 10.3390/biom10081110

**Published:** 2020-07-26

**Authors:** Filippo Pinto e Vairo, Diana Rojas Málaga, Francyne Kubaski, Carolina Fischinger Moura de Souza, Fabiano de Oliveira Poswar, Guilherme Baldo, Roberto Giugliani

**Affiliations:** 1 Center for Individualized Medicine, Mayo Clinic, Rochester, MN 55905, USA; vairo.filippo@mayo.edu; 2 Department of Clinical Genomics, Mayo Clinic, Rochester, MN 55905, USA; 3 Grupo Fleury, São Paulo 04344-070, Brazil; 4 Medical Genetics Service, HCPA, Porto Alegre 90035-903, Brazil; fkubaski@udel.edu (F.K.); cfsouza@hcpa.edu.br (C.F.M.d.S.); fposwar@hcpa.edu.br (F.d.O.P.); rgiugliani@hcpa.edu.br (R.G.); 5 Postgraduate Program in Genetics and Molecular Biology, UFRGS, Porto Alegre 91501-970, Brazil; gbaldo@hcpa.edu.br; 6 Biodiscovery Laboratory, HCPA, Porto Alegre 90035-903, Brazil; 7 INAGEMP, Porto Alegre 90035-004, Brazil; 8 Gene Therapy Center, HCPA, Porto Alegre 90035-903, Brazil; 9 Department of Physiology and Pharmacology, UFRGS, Porto Alegre 90050-170, Brazil; 10 Postgraduate Program in Biological Sciences: Physiology, UFRGS, Porto Alegre 90050-170, Brazil; 11 Department of Genetics, UFRGS, Porto Alegre 91501-970, Brazil

**Keywords:** lysosomal diseases, precision medicine, enzyme replacement therapy, pharmacological chaperones, gene therapy.

## Abstract

Precision medicine (PM) is an emerging approach for disease treatment and prevention that accounts for the individual variability in the genes, environment, and lifestyle of each person. Lysosomal diseases (LDs) are a group of genetic metabolic disorders that include approximately 70 monogenic conditions caused by a defect in lysosomal function. LDs may result from primary lysosomal enzyme deficiencies or impairments in membrane-associated proteins, lysosomal enzyme activators, or modifiers that affect lysosomal function. LDs are heterogeneous disorders, and the phenotype of the affected individual depends on the type of substrate and where it accumulates, which may be impacted by the type of genetic change and residual enzymatic activity. LDs are individually rare, with a combined incidence of approximately 1:4000 individuals. Specific therapies are already available for several LDs, and many more are in development. Early identification may enable disease course prediction and a specific intervention, which is very important for clinical outcome. Driven by advances in omics technology, PM aims to provide the most appropriate management for each patient based on the disease susceptibility or treatment response predictions for specific subgroups. In this review, we focused on the emerging diagnostic technologies that may help to optimize the management of each LD patient and the therapeutic options available, as well as in clinical developments that enable customized approaches to be selected for each subject, according to the principles of PM.

## 1. Introduction

According to the National Institutes of Health (NIH), “precision medicine” (PM) is “an emerging approach for disease treatment and prevention that takes into account individual variability in genes, environment, and lifestyle for each person.” The terms “personalized medicine”, “individualized medicine”, and “precision medicine” have been used interchangeably in recent years; however, precision medicine has been the preferred term since 2015 when the Precision Medicine Initiative (PMI) was launched.

The PMI is a research endeavor funded by the NIH that aims to understand the functional consequences of individual genomic variations and how they interact with the environment to determine the best approach to prevent or treat diseases [1]. PM encompasses the use of advanced diagnostic tools, such as genomic analyses through whole-exome sequencing (WES) or whole-genome sequencing (WGS); other omics, such as metabolomics and epigenomics; advanced imaging; personal and population health information, and big data analytics [2]. Thus, PM refers to the tailoring of the treatment to an individual genetic background, but it does not mean the creation of devices or drugs for only a specific individual. It provides the ability to sort individuals into categories based on their disease susceptibility or predicted response to a treatment. Nonetheless, the term “personalized medicine” may be misleading, suggesting that a unique treatment can be designed for each person, which is not accurate [3].

Lysosomal diseases (LDs) are a group of approximately 70 monogenic disorders caused by a defect in lysosomal function. LDs may result from enzymatic deficiencies (e.g., Gaucher disease (GD)) or impairments in membrane-associated transporters (e.g., cystinosis), enzyme modifiers (e.g., mucolipidosis II and III) or activators (e.g., saposin deficiency) [4]. Although individually rare, the incidence of LDs as a group is estimated to be as high as 1 in 4000 in some countries [5,6]. LDs are heterogeneous disorders, and the phenotype of the affected individual depends on the type of substrate and where it accumulates, as well as the type of genetic change and residual enzymatic activity. Some phenotypes are common to many LDs, such as coarse facies, progressive developmental delay, visceromegaly, and skeletal changes. However, there are disorders that mainly affect the central nervous system (CNS (e.g., neuronal ceroid lipofuscinoses [NCLs])) and others that do not present with primary CNS involvement.

The standard diagnostic work-up for several LDs is based on the clinical presentation; the detection of biomarkers in blood, urine, or cerebrospinal fluid (CSF), and the direct measurement of enzyme activity in leukocytes, plasma, or fibroblasts. Genetic testing is usually performed to confirm or document the biochemical diagnosis but, depending on the disease, might be the only available diagnostic approach.

In the past two decades, pharmaceutical companies have strongly invested in the development of specific therapies for the treatment of LDs, mainly in the form of recombinant enzymes for enzyme replacement therapy (ERT (Table 1)). These medications are usually used intravenously and aim at breaking down the substrates which have accumulated due to an enzymatic deficiency. GD was the first LD to benefit from this approach, with great success. Notably, there is variability in the clinical efficacy due to the severity of the clinical picture, presence of antibodies against the recombinant enzyme, and lack of efficiency in difficult-to-target organs, such as bone, cartilage, and, more importantly, the brain, since the enzyme does not cross the blood–brain barrier (BBB) [7].

Before the development of ERT, hematopoietic stem cell transplantation (HSCT) was the only therapy to treat LDs. It has been used successfully to treat GD and neuronopathic forms of mucopolysaccharidosis (MPS) type I, for instance (Table 1).

Another treatment approach is substrate reduction, which aims to decrease the production of the storage material. There are market-approved drugs in this category for GD and Niemann–Pick type C (NPC) and several clinical and preclinical trials for other diseases, such as Fabry disease (FD), Pompe disease (PD), and GM_2_ gangliosidosis (GM_2_). Regardless of the specific treatment, individuals with LDs usually need a multidisciplinary approach for supportive care to prevent and treat complications, which includes hearing aids, orthoses, speech, respiratory and physical therapies, and psychological support, among others. Importantly, several LDs are targets of researchers and companies interested in gene therapy and genome editing approaches since they are well-characterized monogenic diseases; there are still unmet needs, and even a slight increase in the enzymatic activity could be sufficient to achieve clinical benefits.

The basic concept of PM has been a part of healthcare for many years. Solid organ or bone marrow transplant recipients have always been matched to compatible donors to reduce the risk of complications. For example, in the LD field, ERT dosage has been tailored or even deemed unnecessary. Moreover, hematopoietic stem cell transplantation (HSCT) has been based on the genotype of patients. However, the more comprehensive concept of PM, including novel molecular diagnostic tools and big data analysis, is still relatively limited in medical practice.

In this review, we highlight the advances in the diagnostic approaches and in the development of novel tailored therapies for individuals with LDs.

## 2. Molecular Diagnosis Advances for Lysosomal Diseases

Among the novel technological drivers of PM, next-generation sequencing (NGS) methods have the greatest effect on the diagnostics of LDs since they are becoming more accessible and affordable [17]. NGS applications include the sequencing of a set of specific genomic regions (targeted NGS panel), WES and WGS. These technologies are powerful approaches to overcome the wide clinical and genetic heterogeneity of LDs, allowing the simultaneous screening of several LD-related genes with shorter turnaround times for the final report [18,19].

In the NGS targeted panel, the genes investigated are known to be related to the patient’s phenotype. This approach is heavily dependent on a diagnostic hypothesis that determines which set of genes will be analyzed for each patient, which is disadvantageous when patients with milder or atypical phenotypes are analyzed. In this scenario, a comprehensive gene panel could be helpful, allowing the expansion of the phenotypic spectrum of these disorders. For example, central nervous system involvement is predominant in MPS III, several neuronal ceroid lipofuscinoses, and Tay–Sachs disease, which would justify the inclusion of the associated genes in a neurological-focused panel. Meanwhile, MPS IV-, MPS VI-, or pycnodysostosis-associated genes could be included in a general skeletal dysplasia panel [18]. Whole–exomesequencing (WES) is a standard diagnosisprocedureinmanyplacesandlikewiseisusedtofindnovelgenesassociatedwithrareconditions, such as the newly discovered MPStype (mucopolysaccharidosis–plussyndrome, MPSPS) [20,21], expanding the recognized phenotypic spectrum of known LDs [22,23] and elucidating complex phenotypes [24]. Moreover, several LDs remain undiagnosed after extensive genetic and biochemical investigations due to a wide phenotypic spectrum of nonspecific manifestations or lack of available clinical tests, so WES may be used to identify undiagnosed patients [25]. Although WGS is still not part of a routine clinical diagnosis, it has often been used in a research context to aid in the characterization of complex alterations [26,27]. Notably, ethical aspects are one of the challenges of WES and WGS due to the possibility of identifying variants in genes not related to the main phenotype [28].

Currently, molecular analyses using these three approaches have several roles in LD diagnosis: (1) to confirm the final diagnosis, mainly in milder and atypical cases [29]; (2) to clarify borderline biochemical results in screening and enzymatic assays, as obtained in carriers and pseudodeficiency cases (when enzyme activity is decreased but with no clinical consequences) [30]; (3) to characterize a novel gene associated with a new type of LD, such as the cases of *VPS33A* in MPSPS [21] and *DESG1* in leukodystrophy [31]; (4) for prenatal diagnosis [32,33]; (5) to predict disease severity [34,35]; (6) for the identification of patients with variants amenable to targeted therapy [36,37].

Although next-generation sequencing is revolutionizing LDs diagnosis, the sheer number of distinct conditions, the limited number of patients affected by each rare disease, and the high associated costs represents major challenges for drug development for these diseases.

## 3. Metabolomics as a New Tool for Diagnosis and Monitoring

The term metabolomics was introduced in 2002 to define the deep analysis of small molecules that are part of metabolic pathways by high-throughput analytical methodologies combined with high-end statistical analyses [38,39,40]. Due to the complexity of the interactions of metabolites in our complex systems, these molecules should be properly identified and quantified. The main methods employed for these analyses are nuclear magnetic resonance (NMR) spectroscopy and mass spectrometry (MS), which can be additionally coupled to reduced ion suppression with capillary electrophoresis (CE) for polar charged compounds, gas chromatography (GC) for the analysis of volatile compounds or liquid chromatography (LC) for the analysis of polar and nonpolar compounds [40,41].

Metabolomics can be extremely useful for the diagnosis and monitoring of LDs and can also lead to the discovery of novel biomarkers that can be crucial from diagnosis to treatment follow-up. These molecules can even be used for the discovery of novel LDs [40,42,43].

In 2005, the Human Metabolome Project was launched, aiming to identify connections between genes, diseases, and metabolites to aid the investigation of metabolites associated with inborn errors of metabolism that are reported in the human metabolome database (HMDB) [44,45]. The HMDB is currently the largest database of metabolomics, and it can be used to clarify which metabolic pathway is disturbed for each disorder, enabling the identification and study of key metabolites. A classic example of how metabolomics has aided in the diagnosis and monitoring of LDs is the discovery of globotriaosylsphingosine (lyso-Gb3) as a biomarker for FD [46,47,48,49,50,51].

The two major approaches used in metabolomics studies are based on targeted and untargeted analysis. Targeted metabolomics comprises the analysis of specific metabolites that are usually quantified and compared, leading to the establishment of reference ranges. This panel-based approach can reduce the time of diagnosis for several disorders [51,52,53]. The untargeted approach consists of analyzing all the detectable metabolites (known and unknown) in any type of sample matrix (tissue or biological fluid) to elucidate whether there are any abnormalities that can be correlated with disorders [53,54]. The matrix-assisted laser desorption/ionization-time of flight mass spectrometry (MALDI-TOF) profiling has been widely used for untargeted metabolomics followed by liquid chromatography tandem mass spectrometry [40,55].

The targeted analysis of metabolites is useful in newborn screening (NBS) of LDs (as a primary target, or in a second-tier analysis), as well as to monitor treatment. However, more recently, an untargeted analysis has also been applied to some LDs. Untargeted metabolomics can deepen the understanding of disease mechanisms, allowing discoveries of better biomarkers, new treatment options and personalized therapies. As an example, a metabolome analysis revealed an abnormal polyamine metabolism in the cerebrospinal fluid (CSF) of patients with neuronopathic forms of mucopolysaccharidosis (MPS). Since treatment options for MPS I vary according to the patient’s phenotype, the assessment of these metabolites can help to decide which treatment is more appropriate for each patient [56].

In another study [57] performed in MPS IIIB mice, 231 serum metabolites were altered early in the disease’s natural history. The authors treated mice with gene therapy, and almost 90% of these molecules were corrected. Considering the limitations of current biomarkers, these data show that the metabolites can be used as surrogate markers; their normalization could indicate treatment effectiveness and could be potentially used for the adjustment of doses, for example.

## 4. Next-Generation Treatments

While standard treatments are successful in many instances, there are also certain groups of patients for which these standard approaches are not the best solution. Some alternative therapeutic approaches, such as pharmacological chaperones, gene therapy, and substrate reduction therapy, are in development, with many currently under clinical trials demonstrating great potential (Table 2).

## 5. Small Molecules

Small molecules are low molecular weight synthetic compounds that address the pathophysiological mechanisms of LDs in different manners, such as substrate synthesis inhibition (SSI), the enhancement of enzyme stability, or premature termination codon read-through. These molecules have some advantages over ERT, such as the possibility of being administered orally, the lack of hypersensitive reactions, low manufacturing costs, and more importantly, the ability to cross the BBB [58].

### 5.1. Pharmacogenomics and Small Molecules

Variants in polymorphic genes that code for some of the cytochrome P450 (CYP) enzymes are known to influence the metabolism of certain drugs. These are called pharmacogenes, and the study of how an individual’s genomic profile influences their response to medications is called pharmacogenomics (PGx), which is a core element of PM [59]. Genetic-based drug prescription not only improves the outcome of treatments but also reduces the risk of adverse effects. Individuals with variants that cause less active or inactive alleles are called poor metabolizers. The lack of activity of a pharmacogene may cause an overdose or an increase in the toxicity of a certain medication. On the other hand, ultrarapid metabolizers may experience a lack of efficacy. If the medication is a prodrug (such as clopidogrel), it might not be effective in an individual who is a poor metabolizer or might cause toxicity in someone who is a rapid metabolizer. Notably, over 90% of the population has at least one impactful variant in a pharmacogene, which should prompt a change in dosing or a change in drug prescription [60].

### 5.2. Substrate Synthesis Inhibition

Miglustat (Zavesca^®^, Actelion Pharmaceuticals, Allschwil, Switzerland) is a synthetic analog of D-glucose and was the first market-approved SSI drug for the treatment of GD and NPC [61]. Although miglustat has been shown to reduce the accumulation of glycosphingolipids in animal models of other LDs, it has not been approved for the treatment of patients due to a lack of measurable clinical benefits [62]. Interestingly, PM and PGx became more relevant for the LD field after the approval of eliglustat (Cerdelga™, Sanofi-Genzyme, Cambridge, MA, USA) as a treatment option for GD.

Eliglustat is an oral substrate reduction therapeutic that may be used as a first-line therapy for patients with the nonneuropathic form of GD who are not CYP2D6 ultra-rapid metabolizers [63]. However, eliglustat does not cross the BBB, limiting its effects on the neuropathic forms of the disease. Another example is cysteamine, which is the treatment of choice for nephropathic cystinosis since it delays progression of the renal and extrarenal manifestations and has a strong impact on survival rates [64]. Disease progression in NPC patients has been proven to stabilize with intrathecal 2-hydroxypropyl-β-cyclodextrin (HP-β-CD) since it does not cross the BBB [65]. Recently, drug-loaded nanoparticles were tested in NPC mice and were able to reach the CNS at a higher rate than other organs, making them a potential novel approach to treat patients [66]. Based on the preclinical data regarding the reduction of GAG accumulation, genistein has been tested in different types of MPS with conflicting results but an overall lack of clinical benefit. For MPS III, a phase III, randomized, placebo-controlled, clinical trial of high-dose oral genistein (EudraCT number: 2013-001479-18) failed to meet the primary goals, so there is no support for the use of genistein to treat these patients.

### 5.3. Pharmacological Chaperones

Missense variants may cause protein misfolding, which might lead to premature degradation. Pharmacological chaperones may interact with the mutant enzyme and prevent or delay its degradation. Migalastat (Galafold™, Amicus Therapeutics, Cranbury, NJ, USA) was the first pharmacological chaperone approved for the treatment of adult individuals who have a confirmed diagnosis of FD and a drug-amenable *GLA* missense variant. In addition to migalastat, several compounds have been proven to enhance lysosomal enzyme stability, such as ambroxol [67] and NCG607 [68] for glucocerebrosidase (GCase), progranulin for GCase, cathepsin D, hexosaminidase A [69,70], among others [71]. There are ongoing clinical trials testing chaperones in combination with ERT for PD. Preliminary results show an increase in α-glucosidase activity by two-fold compared with that of ERT alone [72].

### 5.4. Premature Termination Codon Read-Through

Several LDs are caused by nonsense variants leading to premature termination codons (PTCs), resulting in truncated enzymes. PTCs (or stop codons) are often more deleterious than missense variants because they lead to a loss of allele expression. Therefore, individuals with LDs caused by PTCs usually present severe phenotypes. There are drugs being tested on cell lines and animal models that promote the ribosome to read through PTCs and translate a somewhat functional enzyme. For example, chloramphenicol has been shown to enhance alpha-L-iduronidase (IDUA) activity in cell lines of MPS I patients [73], whereas ataluren has shown effects on the galactocerebrosidase activity in Krabbe disease (KB) mice [74] and on palmitoyl-protein thioesterase 1 activity in NCL mice [75]. Based on these examples, a genetic diagnosis for an individual with LD has become mandatory not only to provide adequate family counseling but also to tailor therapeutic management. To date, there is no market-approved LD-specific drug in this category. However, some aminoglycoside and nonaminoglycoside compounds have shown promising results in cell-based studies for cystinosis [76], neuronal ceroid lipofuscinosis, and others [77].

## 6. Next-Generation ERT

While alternative therapeutic approaches involving small molecules and gene therapy are being developed, modifications and additions to the ERT field are being explored to improve the range, efficacy and/or convenience of this modality of treatment. Most of these modifications try to address an important unmet need, which relates to the CNS manifestations of LDs, as the standard intravenous ERT is not able to cross the blood–brain barrier (BBB). Other difficulties of standard ERTs are related to the compliance with weekly or biweekly infusions for life and the low efficacy in some tissues/organs. Precision medicine is intrinsically related to these new alternatives, as it is expected that specific patients will better respond to a specific approach, and the choices will be highly related to the genetic background of each affected individual.

### 6.1. Intrathecal and Intracerebroventricular ERT

To address the neurological manifestations of several LDs, the enzyme should be able to reach the CNS. This does not occur with standard intravenous ERT, as the standard enzyme does not cross the BBB in significant amounts. To physically overcome the BBB, the enzyme may be administered intrathecally (IT) or by intracerebroventricular (ICV) injections. IT-ERT has been attempted for MPS IIIA, but the trial was interrupted due to a lack of efficacy. However, ICV-ERT has already been approved to treat type 2 neuronal ceroid lipofuscinosis–CNL2 (BioMarin Pharmaceuticals). IT-ERT is in clinical development to treat metachromatic leukodystrophy-MLD (Takeda). For severe MPS II, both IT (Takeda) and ICV (Green Cross) ERT administrations are being explored [78].

### 6.2. Intravenous ERT that Bypasses the BBB

Another approach to address the neurological manifestations of LDs is to use an alternative formulation of the enzyme, which may be provided as a “fusion protein” (also known as a “Trojan horse”). These fusion proteins combine the therapeutic enzyme with an antibody that is able to be recognized by a specific receptor and allowed to cross the BBB. Two examples of this approach are AGT-181, a fusion protein combining IDUA with an antibody that binds to the insulin receptor at the BBB for the treatment of MPS I (Armagen Technologies), and JR-141, a fusion protein combining iduronate sulfatase with an antibody that binds the transferrin receptor in the BBB for the treatment of MPS II (JCR Pharmaceuticals). Improvements in neurodevelopmental tests and neuroimaging biomarkers were reported with the use of AGT-181 in MPS I [79], and a significant decrease in heparan sulfate in the CSF was observed with JR-141 [80]. Other LD targets already disclosed are MPS I, MPS IIIA, MPS IIIB, MPS VII, and PD (JCR Pharmaceuticals), whereas MPS II and MPS IIIA are targeted by an alternative similar approach (Denali Therapeutics).

### 6.3. Intravenous ERT with Extended Half-Life

Intravenous ERT has a very short half-life in plasma, and infusions should be provided every week or every two weeks, depending on the disease. Some modifications introduced in the recombinant enzyme could extend the half-life and potentially enable longer intervals between infusions, which could represent a significant improvement in the convenience for the patient, as well as a reduction in costs for the health care system. A PEGylated formulation of alfa-galactosidase A is in development (Protalix BioTherapeutics) to treat FD, and the possibility of monthly administration is being explored. The investigators claim that the formulation could potentially have further advantages, in addition to an improved convenience [81].

### 6.4. ERT Administered via Encapsulated Cells Implanted in the Patient

The lack of convenience of weekly or biweekly infusions for life is also being tentatively addressed by the implantation of capsules containing genetically modified cells. The properties of these capsules prevent patient antibodies from attacking the cells and simultaneously allow the cells to obtain nutrients and produce the enzymes which leave the capsules to reach the tissues and organs [82]. The main advantages of this strategy over standard intravenous ERTs relate to convenience, as the patient will not need to receive regular infusions, and to the continuous release of enzyme (instead of weekly or biweekly pulses of enzyme). The disadvantages are the small surgery needed to implant the encapsulated cells, which will probably need to be repeated from time to time, and the fact that, if the cells produce a standard enzyme, it will not be able to cross the BBB. This strategy, which is being developed for FD and MPS I (Sigilon Therapeutics), could potentially address other LDs.

### 6.5. Intravenous ERT Combined with Oral Pharmacological Chaperones

A pharmacological chaperone (migalastat) was developed and already approved to treat patients with FD who harbor amenable mutations, as an alternative to ERT. The same product is being clinically tested in combination with ERT in patients with PD (Amicus Therapeutics). In this case, the chaperone would boost the enzyme activity, which is expected to improve outcomes [83].

### 6.6. Intravenous ERT for Other LDs

The number of LDs that have a specific ERT available is continuously increasing. Acid sphingomyelinase deficiency (ASMD) has acute and chronic forms. Chronic ASMD (formerly known as Niemann–Pick type B disease) has a presentation somewhat similar to that of GD type I with hepatomegaly, splenomegaly, anemia, and thrombocytopenia as well as interstitial lung disease. An intravenous ERT for chronic ASMD (olipudase alfa, Sanofi S.A.) is already in an advanced stage of clinical development [84].

## 7. Gene Therapy/Genome Editing

Gene therapies for LDs are advancing rapidly. Although not all of them, most LDs present brain involvement, stimulating the development of therapies targeting this organ [85].

There are two gene therapy approaches for brain involvement in LDs. In one case, the vector carrying the therapeutic gene is administered in vivo, either by in situ administration or by an intravenous injection of a vector with a tropism for brain cells [71,86]. The latter can also be used for other LDs without brain involvement by targeting the vector to other organs [87]. One example of a clinical trial using in vivo gene therapy for an LD includes the intraparenchymal injections of a recombinant adeno-associated viral vector serotype 2/5 (rAAV2/5) in children with mucopolysaccharidosis type IIIB [88].

Another possible scenario is hematopoietic stem cell (HSC)-targeted gene therapy. The rationale for this approach is that microglial cells originate from the differentiation of hematopoietic precursor cells. Thus, HSCs can be harvested, corrected ex vivo, and infused back into the patient. Gene-corrected HSCs repopulate the bone marrow and eventually migrate into the brain and differentiate into microglial cells. These cells then secrete the enzyme and cross-correct neurons and other brain cells. Eight out of nine children with metachromatic leukodystrophy who were submitted to an ex vivo gene therapy protocol experienced the prevention of disease onset or a halted disease progression [89], showing the potential of this approach.

Although CRISPR-Cas9 genome editing has been tested in preclinical trials for different LDs [90], so far, only zinc finger-mediated gene editing has been applied in a trial for an LD—Hunter syndrome [91]. The trial is still ongoing, and no efficacy can be inferred at this point, but preliminary results showed no serious adverse effects using this approach.

## 8. Antisense Oligonucleotide Therapy

Antisense oligonucleotide (ASO) technology has emerged as a powerful therapeutic alternative for the treatment of genetic disorders by targeting cellular RNA and controlling gene expression through several distinct mechanisms. Novel chemical modifications of single-stranded deoxynucleotides allowed the development of next-generation ASOs with enhanced pharmacological properties. This is reflected in the fact that in the past few years, ASO therapies were approved for the treatment of spinal muscular atrophy and Duchenne muscular dystrophy [92].

ASO-based therapies for LDs are focused mainly on restoring the normal splicing of mutated transcripts. At least 600 mutations that affect precursor mRNA (pre-mRNA) splicing have been described in patients with LDs. Most are private variants, but a common splicing variant accounts for up to 70% of the pathogenic alleles for PD, FD, mucolipidosis type II/III, and Tay–Sachs disease, representing excellent candidates for this type of approach [93].

The first attempt to develop an ASO therapy for an LD was performed in a cellular model of NPC disease carrying a variant that creates a cryptic donor splice site, resulting in the incorporation of 194 bp of intron nine as a new exon (pseudoexon) [94]. The strategy was able to restore normal splicing. Since then, several other studies have reported this approach in the treatment of late-onset PD caused by the c.-32-13T>G variant present in 40–70% of the alleles [95] and for MPS type II [96].

Recently, an unprecedented example of precision medicine for LD was published. Kim et al. [26] reported the discovery, development, and administration of milasen, a splice-modulating antisense oligonucleotide drug tailored to a single patient. Mila was a six-year-old girl with CLN7 neuronal ceroid lipofuscinosis, which is a fatal neurodegenerative condition. After a WGS analysis, a known pathogenic variant was found in trans with a novel insertion of an SVA (SINE-VNTR-Alu) retrotransposon in *MFSD8*. The SVA causes missplicing of the MFSD8 mRNA and leads to premature translational termination. The researchers customized an ASO that blocked the cryptic splice-acceptor site, increased the ratio of normal to mutant mRNA and restored the *MFSD8* expression. Moreover, there was a decrease in intracellular vacuolization in the patient’s fibroblasts. Due to clinical urgency and promising in vitro results, the Food and Drug Administration (FDA)-approved milasen to be administered by intrathecal injection to this single patient in ascending doses. As a result, seizures decreased from 30 episodes per day lasting 1 to 2 min to just a few episodes lasting a few seconds. Remarkably, the path from the identification of the variants to the development of the tailored drug and clinical deployment occurred in less than 12 months [26].

## 9. Combination of Therapies

When the first therapies for LDs were announced, they were focused on correcting the primary enzymatic defect and some of the most striking symptoms. However, LDs are multisystem diseases with difficult-to-treat affected organs, so monotherapies frequently do not improve all symptoms. Similar to multifactorial diseases, a combination of therapies may be necessary to achieve the best therapeutic response. For example, ERT has limited success in treating bone, cartilage, and the heart in MPS I, II, and VI. In patients with PD in advanced stages of the disease, skeletal muscle impairment remains refractory to ERT [97]. 

Although CNS administration of ERT, HSCT, or gene therapy circumvents the BBB, the widespread involvement of multiple brain regions in LDs and the inability of treatments to diffuse freely throughout the parenchyma result in only partial correction of CNS pathology [98].

There are combined strategies in clinical and preclinical trials for LDs. The most common combination therapy for a LDs leverages the success of ERT by coupling it with chaperones, SSIs, gene therapy, or HSCT. (i) ERT and HSCT are therapeutic options to halt the disease progression but are not curative. HSCT is the standard of care for children with severe MPS I, and ERT is typically initiated prior to transplantation to improve somatic symptoms and the ability to tolerate conditioning and transplants. Despite HSCT, growth failure with short stature along with musculoskeletal complications remains a prominent manifestation of MPS IH. ERT in combination with HSCT also enhances cognitive outcomes. The real benefit of ERT post-HSCT is still under evaluation [99,100]. (ii) For SSI + ERT, the oral selective glucosylceramide synthase inhibitor (venglustat) is under investigation for the treatment of GD type 3. Combined with ERT (imiglucerase), it is being assessed in the phase 2 LEAP trial in 11 patients aged ≥18 years to evaluate neurological outcomes since venglustat has been shown to cross the BBB [101]. (iii) For gene therapy and HSCT, HSCT synergized with CNS-directed AAV-mediated ERT reduced storage, decreased neuroinflammation, improved motor deficits, and dramatically improved the lifespan of an NCL animal model [102]. (iv) For CNS-directed gene therapy, systemic SSI, and HSCT, this combination modality has been studied in a preclinical mouse model of KD. Simultaneously, treating multiple pathogenic targets resulted in an unprecedented increase in life span with improved motor function, persistent GALC expression, nearly normal psychosine levels, and decreased neuroinflammation [103].

Although combinations of therapies that involve gene therapy or genome editing are still not available for patients, the data from preclinical trials are very promising. Understanding the pathophysiology of LDs and identifying the secondary mechanisms involved in the pathogenesis, such as ER stress, altered lipid trafficking, autophagy impairment, inflammation, and altered calcium homeostasis, will aid in the development of personalized therapies for the diverse symptoms of LDs [13].

## 10. Concluding Remarks

The goal of PM is to use biological knowledge and health information to predict disease risk, understand disease pathophysiology, identify disease subcategories, improve diagnoses, and provide tailored treatment strategies to achieve the best possible outcomes. In the LD field, Mila´s case mentioned above perfectly illustrates how recently developed technologies, such as whole-genome sequencing and antisense approaches, allowed the delineation of pathways for ultrapersonalized medicine.

Although PM has the potential to profoundly improve LD management, the required advances will take some time to become routine. For example, the collection and analysis of patients’ data will be invaluable for PM to reach its potential in this field. Clinical and research laboratories often create biorepositories, whose power depends on the number and types of samples. It is a great challenge to develop biorepositories for LDs since there are limited numbers of individuals reported due to rarity or underdiagnoses. However, this challenge might be overcome by national and international collaborative efforts and multicenter data sharing, data collection standardization, and patient education on the benefits of sharing experiences and samples.

Recent advances in omics are already being used and have been instrumental in directing clinical decision making. The integration of multiomics data has the potential to drive real changes in PM and will be crucial to understanding the biological and cellular mechanisms of LD-causing variants and developing novel approaches for LD treatment in an individualized manner. However, the analysis and interpretation of omics data is challenging, especially because they are highly complex and voluminous; for this purpose, the development of novel bioinformatics methods and databases will be necessary.

Finally, this article intended to highlight the novel diagnosis and treatment modalities for LDs based on a PM approach. Even though novel therapies such as antisense oligonucleotides and genome editing are still in their early stages, they have already shown promising results in the clinical setting, suggesting that they are real possibility for several LDs in the near future.

## Figures and Tables

**Table 1:** Market-approved enzyme replacement therapies (ERTs) and hematopoietic stem cell transplantation (HSCT) outcomes.

Disease	Product	Manufacturer	FDA or EMA* Approval	Prescribing Information	Comments on HSCT
Alpha-Mannosidosis	Lamzede^®^	Chiesi	2018*	https://www.ema.europa.eu/en/documents/product-information/lamzede-epar-product-information_en.pdf	Effective, maybe an alternative to ERT [8]
Fabry disease	Replagal^®^	Takeda/Shire	2001	https://www.ema.europa.eu/en/documents/product-information/replagal-epar-product-information_en.pdf	Not effective
	Fabrazyme^®^	Sanofi/Genzyme	2001	www.fabrazyme.com/hcp/pi/fz_us_hc_pi.pdf	
	Fabagal^®^#	ISU ABXIS	2014#	http://www.abxis.com/eng/product/doc_fabagal.pdf	
Gaucher type I	Cerezyme^®^*	Sanofi/Genzyme	1994	www.cerezyme.com/~/media/Files/CerezymeUS/pdf/cerezyme_pi.pdf	Effective, maybe an alternative to ERT [9]
	Velaglucerase alfa	Takeda/Shire	2010	www.accessdata.fda.gov/drugsatfda_docs/label/2010/022575lbl.pdf	
	Elelyso™	Pfizer/Protalix	2012	www.elelyso.com/pdf/ELELYSO_Prescribing_Information.pdf	
	Abcertin^®^#	ISU ABXIS	2012#	http://www.abxis.com/eng/product/doc_abcertin.pdf	
Lysosomal acid lipase deficiency	Kanuma™	Alexion	2015	https://kanuma.com/hcp	High mortality rate due to transplant related complications, liver failure or sinusoidal obstruction syndrome [10]
Mucopolysaccharidosis I	Aldurazyme^®^	Sanofi/Genzyme	2003	www.aldurazyme.com/pdf/az_us_hc_pi.pdf	Effective, maybe an alternative to ERT; recommended for patients with the Hurler phenotype if performed early [11]
Mucopolysaccharidosis II	Elaprase^®^	Takeda/Shire	2006	www.elaprase.com/pdf/Elaprase_US_PI_v6.pdf	Effective if performed in early disease stages before irreversible disease manifestations have occurred [12,13]
	Hunterase^®^	GC Pharma/Nanolek LLC	2012#	https://www.nanolek.ru/en/product/biotekhnologicheskie/khanteraza/	
Mucopolysaccharidosis IVA	Vimizin^®^	BioMarin	2014	https://vimizim.com/hcp/prescribing-information/	Reported in a few cases, may be an alternative treatment if performed early [14]
Mucopolysaccharidosis VI	Naglazyme™	BioMarin	2005	www.naglazyme.com/en/documents/Naglazyme_Prescribing_Information.pdf	Effective, maybe an alternative to ERT [15]
Mucopolysaccharidosis type VII	Mepsevii™	Ultragenyx	2017	https://www.accessdata.fda.gov/drugsatfda_docs/label/2017/761047s000lbl.pdf	Reported in a few cases, may be an alternative treatment if performed early [16]
Neuronal ceroid lipofuscinosis type 2	Brineura™	BioMarin	2017	https://www.brineura.com/wp-content/themes/jupiter-child/assets/pdfs/resources/Brineura-Dosing-and-Administration-Guide.pdf	Not effective
Pompe disease	Myozyme^®^	Sanofi/Genzyme	2006	www.accessdata.fda.gov/drugsatfda_docs/label;/2008/125141_74lbl.pdf	Not effective
	Lumizyme^®^	Sanofi/Genzyme	2010	www.accessdata.fda.gov/drugsatfda_docs/label/2010/125291lbl.pdf	

# approved in South Korea. * Ceredase was made from placenta and it was the precursor of Cerezyme^®^.

**Table 2:** Active clinical trials for lysosomal diseases (as of June, 2020).

Disease	Treatment	Product	Expected outcomes	Cons	Status	EudraCT number	NCT Number	Sponsor
Acid Sphingomyelinase Deficiency	Intravenous enzyme replacement therapy	Olipudase alfa	Overall disease improvement	Efficacy to be determined	Terminated	n/a	NCT00410566	Genzyme, a Sanofi Company
	Intrathecal administration of cell therapy	Umbilical cord blood-derived oligodendrocyte-like cells	Rapid delivery of donor cells in the CNS; constant enzyme secretion; one-time treatment	Conditioning side-effects; safety and efficacy still to be determined	Recruiting	n/a	NCT02254863	Duke University, Durham, NC
Alpha-mannosidosis	Intrathecal administration of cell therapy	Umbilical cord blood-derived oligodendrocyte-like cells	Rapid delivery of donor cells in the CNS; constant enzyme secretion; one-time treatment	Conditioning side-effects; safety and efficacy still to be determined	Recruiting	n/a	NCT02254863	Duke University, Durham, NC
Aspartylglucosaminuria	Chaperone therapy	Betaine	Overall disease improvement	Efficacy and safety to be determined	Ongoing	2017-000645-48	n/a	Orphan Europe SARL
Cystinosis	Stop codon read-through	ELX-02	Overall disease improvement	Efficacy and safety to be determined	Terminated	n/a	NCT04069260	Eloxx Pharmaceuticals, Inc.
	ex vivo gene therapy	Lentiviral vector (CTNS-RD-04)	Overall disease improvement	Efficacy and safety to be determined	Recruiting	n/a	NCT03897361	University of California, San Diego
Danon Disease	Gene therapy	AAV9 vector (RP-A501)	Overall disease improvement	Efficacy and safety to be determined	Recruiting	n/a	NCT03882437	Rocket Pharmaceuticals Inc.
Fabry disease	Liver directed gene therapy	AAV Vector (FLT190)	One-time treatment; broad enzyme distribution	Efficacy to be determined	Recruiting	n/a	NCT04040049	Freeline Therapeutics
	Gene therapy	AAV 2/6 vector (ST-920)	One-time treatment; broad enzyme distribution	Efficacy to be determined	Recruiting	n/a	NCT04046224	Sangamo Therapeutics
	Substrate reduction therapy combined with enzyme replacement therapy	Venglustat + agalsidase beta	Overall disease improvement	Efficacy and safety to be determined	Completed	n/a	NCT02228460	Genzyme, a Sanofi Company
	Enzyme replacement therapy	Pegunigalsidase alfa	Overall disease improvement	Efficacy and safety to be determined	Active, not recruiting	n/a	NCT03018730	Protalix
	Substrate reduction therapy combined with enzyme replacement therapy	Lucerastat+ Fabrazymeor Replagal	Overall disease improvement	Efficacy and safety to be determined	Completed	n/a	NCT02930655	Idorsia Pharmaceuticals Ltd.
	ex vivo gene therapy	Lentiviral vector (AVR-RD-01)	One-time treatment; broad enzyme distribution; less immune response due to the autologous process	Efficacy to be determined	Recruiting	n/a	NCT03454893	AvroBio
Gaucher disease	Chaperone therapy	Ambroxol	Overall disease improvement due to higher enzyme levels	Efficacy and safety to be determined	Recruiting	n/a	NCT03950050	Shaare Zedek Medical Center
	Substrate reduction therapy combined with enzyme replacement therapy	Venglustat + imiglucerase	Overall disease improvement	Efficacy and safety to be determined	Recruiting	n/a	NCT02843035	Genzyme, a Sanofi Company
	ex vivo gene therapy	Lentiviral vector (AVR-RD-02)	Overall disease improvement	Efficacy and safety to be determined	Recruiting	n/a	NCT04145037	AvroBio
Krabbe disease	Intrathecal administration of cell therapy	Umbilical cord blood-derived oligodendrocyte-like cells	Rapid delivery of donor cells in the CNS; constant enzyme secretion; one-time treatment	Conditioning side-effects; safety and efficacy still to be determined	Recruiting	n/a	NCT02254863	Duke University, Durham, NC
GM1 gangliosidosis	Intracisternal gene therapy	AAV9 vector (LYS-GM101)	Overall disease improvement	Efficacy to be determined	Not yet recruiting	n/a	NCT04273269	Lysogene
	Intracisternal gene therapy	AAVhu68 (PBGM01)	Overall disease improvement	Efficacy and safety to be determined	Not yet recruiting	n/a	n/a	PassageBio
	Intravenous gene therapy	AAV9 vector (AAV9-GLB1)	Overall disease improvement	Efficacy to be determined	Recruiting	n/a	NCT03952637	National Human Genome Research Institute
GM2 gangliosidosis	Substrate reduction therapy	Miglustat	Overall disease improvement	Efficacy to be determined	Recruiting	n/a	NCT03822013	Tehran University of Medical Sciences
	Substrate reduction therapy	Venglugstat			Recruiting	n/a	NCT04221451	Genzyme, a Sanofi Company
	Intrathecal gene therapy	rAAVrh8-HEXA/B	Overall disease improvement	Efficacy and safety to be determined	not yet recruiting	n/a	n/a	Axovant
	Intrathecal administration of cell therapy	Umbilical cord blood-derived oligodendrocyte-like cells	Rapid delivery of donor cells in the CNS; constant enzyme secretion; one-time treatment	Conditioning side-effects; safety and efficacy still to be determined	Recruiting	n/a	NCT02254863	Duke University, Durham, NC
Metachromatic leukodystrophy	Intrathecal enzyme replacement therapy	SHP 611	Overall disease improvement	Efficacy to be determined	Recruiting	2018-003291-12	NCT03771898	Takeda
	Hematopoieitic stem cell gene therapy	Lentiviral vector (OTL-200)	Improvement in enzyme levels with sulfatide storage reduction; able to prevent disease if administered in presymptomatic patients; no signs of genotoxicity.	It might not be able to rescue progression in symptomatic patients; long-term follow up is needed to determine possible complications	Active, not recruiting	n/a	NCT01560182	Orchard Therapeutics
	Intrathecal administration of cell therapy	Umbilical cord blood-derived oligodendrocyte-like cells	Rapid delivery of donor cells in the CNS; constant enzyme secretion; one-time treatment	Conditioning side-effects; safety and efficacy still to be determined	Recruiting	n/a	NCT02254863	Duke University, Durham, NC
Mucopolysaccharidosis type I	Enzyme replacement therapy with fusion protein	Valanafusp alfa	Improvement in enzyme levels with reduction in GAG storage in urine, plasma and CSF; drug likely penetrates the BBB	Immune responses that can possibly neutralize the enzyme; efficacy to be determined	Completed	n/a	NCT03053089	ArmaGen, Inc
	Autologous CD34+ HSCT transduced ex vivo gene therapy	Lentiviral vector	Overall disease improvement due to higher enzyme levels	Efficacy and safety to be determined	Recruiting	n/a	NCT03488394	IRCCS San Raffaele
	Enzyme replacement therapy with fusion protein	JR-171	Improvement in enzyme levels with reduction in GAG storage in urine, plasma and CSF; drug likely penetrates the BBB	Immune responses that can possibly neutralize the enzyme; efficacy to be determined	Not yet recruiting	n/a	NCT04227600	JCR Pharmaceuticals Co., Ltd.
	Intracisternal gene therapy	AAV9 vector (RGX-111)	Improvement in enzyme levels with reduction in GAG storage; improvement in CNS	Efficacy to be determined	Recruiting	n/a	NCT03580083	Regenxbio Inc.
Mucopolysaccharidosis type II	Intracisternal gene therapyB32:I38	AAV9 vector (RGX-121)	Improvement in enzyme levels with reduction in GAG storage in urine, plasma and CSF; drug likely penetrates the BBB	Efficacy to be determined	Recruiting	n/a	NCT03566043	Regenxbio Inc.
	Intrathecal enzyme replacement therapy	Idursulfase	Improvement in enzyme levels with reduction in GAG storage in the CSF; improvement in neurological impairment	Efficacy and safety to be determined	Completed	n/a	NCT00920647	Takeda
	Intracerebroventricular enzyme replacement therapy	Idursulfase beta	Overall disease improvement	Efficacy and safety to be determined	Completed	n/a	NCT01645189	GC Pharma
	Enzyme replacement therapy with fusion protein	DNL310	Overall disease improvement	Efficacy and safety to be determined	Not yet recruiting	n/a	NCT04251026	Denali Therapeutics Inc.
	Enzyme replacement therapy with fusion protein	JR-141	Improvement in enzyme levels with reduction in GAG storage in urine, plasma and CSF; drug likely penetrates the BBB	Immune responses that can possibly neutralize the enzyme; efficacy to be determined	Enrolling by invitation	n/a	NCT04348136	JCR Pharmaceuticals Co., Ltd.
	Genome editing	SB-913	One-time treatment; broad enzyme distribution	Immune responses that can possibly neutralize the enzyme; efficacy to be determined	Active, not recruiting	n/a	NCT03041324	Sangamo Therapeutics
	Intrathecal administration of cell therapy**	Umbilical cord blood-derived oligodendrocyte-like cells	Rapid delivery of donor cells in the CNS; constant enzyme secretion; one-time treatment	Conditioning side-effects; safety and efficacy still to be determined	Recruiting	n/a	NCT02254863	Duke University, Durham, NC
Mucopolysaccharidosis type IIIA	Autologous CD34+ HSCT transduced ex vivo gene therapy	Lentiviral vector	One-time treatment; broad enzyme distribution	Efficacy to be determined	Recruiting	n/a	NCT04201405	University of Manchester
	Intravenous gene therapy	AAV09 vector (ABO-102)	Leads to sustained enzyme production in the brain, likely to be one-time treatment, well tolerated	Possible immune response; efficacy still under testing; long-term follow up is needed to determine possible complications	Recruiting	n/a	NCT04088734	Abeona Therapeutics, Inc
	Intracerebral gene therapy	AAV 10 vector (LYS-SAF302)			Recruiting	n/a	NCT03612869	Lysogene
	Intrathecal administration of cell therapy	Umbilical cord blood-derived oligodendrocyte-like cells	Rapid delivery of donor cells in the CNS; constant enzyme secretion; one-time treatment	Conditioning side-effects; safety and efficacy still to be determined	Recruiting	n/a	NCT02254863	Duke University, Durham, NC
Mucopolysaccharidosis type IIIB	Intracerebroventricular enzyme replacement therapy	AX 250	Overall disease improvement	Efficacy and safety to be determined	enrolling by invitation	n/a	NCT03784287	Allievex Corporation
	Intravenous gene therapy with adeno-associated virus (AAV)	AAV9 vector (rAAV9.CMV.hNAGLU)	Overall disease improvement	Efficacy to be determined	Recruiting	n/a	NCT03315182	Abeona Therapeutics, Inc
	Intracerebral gene therapy	AAV2/5 vector (rAAV2/5-hNaGlu)	Leads to sustained enzyme production in the brain, likely to be one-time treatment, well tolerated	Possible immune response; efficacy still under testing; long-term follow up is needed to determine possible complications	Completed	2012-000856-33	n/a	Institut Pasteur
	Intrathecal administration of cell therapy	Umbilical cord blood-derived oligodendrocyte-like cells	Rapid delivery of donor cells in the CNS; constant enzyme secretion; one-time treatment	Conditioning side-effects; safety and efficacy still to be determined	Recruiting	n/a	NCT02254863	Duke University, Durham, NC
Mucopolysaccharidosis type IVA	Cellular signaling pathway inhibition	Losartan	Improvement on cardiac impairment	Efficacy and safety to be determined	Recruiting	n/a	NCT03632213	Hospital de Clinicas de Porto Alegre
Mucopolysaccharidosis type VI	Substrate reduction therapy	Odiparcil	Overall disease improvement	Efficacy and safety to be determined	Completed	n/a	NCT03370653	Inventiva Pharma
	Cellular signaling pathway inhibition	Losartan	Improvement on cardiac impairment	Efficacy and safety to be determined	Recruiting	n/a	NCT03632213	Hospital de Clinicas de Porto Alegre
	Liver directed gene therapy	AAV2/8 vector (AAV2/8.TBG.hARSB)	Overall disease improvement	Efficacy to be determined	Recruiting	n/a	NCT03173521	Fondazione Telethon
Neuronal Ceroid Lipofuscinosis type 2 (CLN2)	Intracerebral gene therapy	AAV vector (AAVrh.10CUhCLN2)	Overall disease improvement	Efficacy to be determined	Active, not recruiting	n/a	NCT01414985	Weill Medical College of Cornell University
Neuronal Ceroid Lipofuscinosis type 3 (CLN3)	Intrathecal gene therapy	AAV9 vector (AT-GTX-502)	Overall disease improvement	Efficacy to be determined	Active, not recruiting	n/a	NCT03770572	Amicus Therapeutics
Neuronal Ceroid Lipofuscinosis type 6 (CLN6)	Intrathecal gene therapy	AAV9 vector (AT-GTX-501)	Overall disease improvement	Efficacy to be determined	Active, not recruiting	n/a	NCT02725580	Amicus Therapeutics
Niemann-pick type C	Intrathecal administration	2-Hydroxypropyl-Beta-Cyclodextrin	Improvement of liver symptoms	Efficacy and safety to be determined	Recruiting	n/a	NCT03471143	Washington University School of Medicine
Pompe disease	Intravenous gene therapy	AAV8 vector (AT845)	Improvement in respiratory function	Efficacy to be determined	Not yet recruiting	n/a	NCT04174105	Audentes Therapeutics
	Intravenous gene therapy	AAV2/8 vector (AAV2/8LSPhGAA)	Improvement in respiratory function	Efficacy to be determined	Recruiting	n/a	NCT03533673	Asklepios Biopharmaceutical, Inc.
	Chaperone + enzyme replacement therapy	AT2221 + ATB200+ alglucosidase alfa	Overall disease improvement	Efficacy to be determined	Ongoing	n/a	NCT03729362	Amicus Therapeutics
	Diaphragm delivery gene therapy	AAV 1 vector (rAAV1-CMV-GAA)	Improvement in respiratory function	Limited results in inspiratory pressure; immune responses; long-term follow up is needed to determine possible complications	Completed	n/a	NCT00976352	University of Florida

## References

[1] Pinto E.V.F., Lazaridis K.N. (2019). Individualized medicine comes to the liver clinic. J. Hepatol..

[2] Hou Y.C., Yu H.C., Martin R., Cirulli E.T., Schenker-Ahmed N.M., Hicks M., Cohen I.V., Jönsson T.J., Heister R., Napier L. (2020). Precision medicine integrating whole-genome sequencing, comprehensive metabolomics, and advanced imaging. Proc. Natl. Acad. Sci. USA.

[3] (2011). Toward Precision Medicine: Building a Knowledge Network for Biomedical Research and a New Taxonomy of Disease.

[4] Platt F.M., d’Azzo A., Davidson B.L., Neufeld E.F., Tifft C.J. (2018). Lysosomal storage diseases. Nat. Rev. Dis. Primers.

[5] Fuller M., Meikle P.J., Hopwood J.J., Mehta A., Beck M., Sunder-Plassmann G. (2006). Epidemiology of lysosomal storage diseases: An overview. Fabry Disease: Perspectives from 5 Years of FOS.

[6] Giugliani R., Federhen A., Michelin-Tirelli K., Riegel M., Burin M. (2017). Relative frequency and estimated minimal frequency of Lysosomal Storage Diseases in Brazil: Report from a Reference Laboratory. Genet. Mol. Biol..

[7] Beck M. (2018). Treatment strategies for lysosomal storage disorders. Dev. Med. Child Neurol..

[8] Mynarek M., Tolar J., Albert M.H., Escolar M.L., Boelens J.J., Cowan M.J., Finnegan N., Glomstein A., Jacobsohn D.A., Kühl J.S. (2012). Allogeneic Hematopoietic SCT for Alpha-Mannosidosis: An Analysis of 17 Patients. Bone Marrow Transplant..

[9] Somaraju U.R., Tadepalli K. (2017). ‘Cochrane Database of Systematic Reviews Hematopoietic Stem Cell Transplantation for Gaucher Disease (Review) Hematopoietic Stem Cell Transplantation for Gaucher Disease. Hematop. Stem Cell Transplant. Gauch. Dis..

[10] Gramatges M.M., Dvorak C.C., Regula D.P., Enns G.M., Weinberg K., Agarwal R. (2009). Pathological Evidence of Wolman’s Disease Following Hematopoietic Stem Cell Transplantation despite Correction of Lysosomal Acid Lipase Activity. Bone Marrow Transplant..

[11] Aldenhoven M., Van Den Broek B.T.A., Wynn R.F., O’Meara A., Veys P., Rovelli A., Jones S.A., Parini R., Van Hasselt P.M., Renard M. (2017). Quality of Life of Hurler Syndrome Patients after Successful Hematopoietic Stem Cell Transplantation. Blood Adv..

[12] Barth A.L., Horovitz D.D.G. (2018). Hematopoietic Stem Cell Transplantation in Mucopolysaccharidosis Type II. J. Inborn Errors Metab. Screen..

[13] Barth L., de Magalhães T.S.P.C., Reis A.B.R., de Oliveira M.L., Scalco F.B., Cavalcanti N.C., Silva D.S.E., Torres D.A., Costa A.A.P., Bonfim C. (2017). Early Hematopoietic Stem Cell Transplantation in a Patient with Severe Mucopolysaccharidosis II: A 7 Years Follow-Up. Mol. Genet. Metab. Rep..

[14] Yabe H., Tanaka A., Chinen Y., Kato S., Sawamoto K., Yasuda E., Shintaku H., Suzuki Y., Orii T., Tomatsu S. (2016). Hematopoietic Stem Cell Transplantation for Morquio A Syndrome. Mol. Genet. Metab..

[15] Turbeville S., Nicely H., Douglas Rizzo J., Pedersen T.L., Orchard P.J., Horwitz M.E., Horwitz E.M., Veys P., Bonfim C., Al-Seraihy A. (2011). Clinical Outcomes Following Hematopoietic Stem Cell Transplantation for the Treatment of Mucopolysaccharidosis VI. Mol. Genet. Metab..

[16] Orii K., Suzuki Y., Tomatsu S., Orii T., Fukao T. (2020). Long-Term Follow-up Posthematopoietic Stem Cell Transplantation in a Japanese Patient with Type-VII Mucopolysaccharidosis. Diagnostics.

[17] Jameson J.L., Longo D.L. (2015). Precision medicine--personalized, problematic, and promising. N. Engl. J. Med..

[18] Brusius-Facchin A.C., Rojas Malaga D., Leistner-Segal S., Giugliani R. (2018). Recent advances in molecular testing to improve early diagnosis in children with mucopolysaccharidoses. Expert Rev. Mol. Diagn..

[19] Nashabat M., Al-Khenaizan S., Alfadhel M. (2019). Report of a Case that Expands the Phenotype of Infantile Krabbe Disease. Am. J. Case Rep..

[20] Kondo H., Maksimova N., Otomo T., Kato H., Imai A., Asano Y., Kobayashi K., Nojima S., Nakaya A., Hamada Y. (2017). Mutation in VPS33A affects metabolism of glycosaminoglycans: A new type of mucopolysaccharidosis with severe systemic symptoms. Hum. Mol. Genet..

[21] Dursun A., Yalnizoglu D., Gerdan O.F., Yucel-Yilmaz D., Sagiroglu M.S., Yuksel B., Gucer S., Sivri S., Ozgul R.K. (2017). A probable new syndrome with the storage disease phenotype caused by the VPS33A gene mutation. Clin. Dysmorphol..

[22] Nikkel S.M., Huang L., Lachman R., Beaulieu C.L., Schwartzentruber J., Majewski J., Geraghty M.T., Boycott K.M., Consortium F.C. (2014). Whole-exome sequencing expands the phenotype of Hunter syndrome. Clin. Genet..

[23] Zeng Q., Fan Y., Wang L., Huang Z., Gu X., Yu Y. (2017). Molecular defects identified by whole exome sequencing in a child with atypical mucopolysaccharidosis IIIB. J. Pediatr. Endocrinol. Metab..

[24] Kaissi A.A., Hofstaetter J., Weigel G., Grill F., Ganger R., Kircher S.G. (2016). The constellation of skeletal deformities in a family with mixed types of mucopolysaccharidoses: Case report. Medicine.

[25] Vairo F.P., Boczek N.J., Cousin M.A., Kaiwar C., Blackburn P.R., Conboy E., Lanpher B.C., Gavrilova R.H., Pichurin P.N., Lazaridis K.N. (2017). The prevalence of diseases caused by lysosome-related genes in a cohort of undiagnosed patients. Mol. Genet. Metab. Rep..

[26] Kim J., Hu C., Moufawad El Achkar C., Black L.E., Douville J., Larson A., Pendergast M.K., Goldkind S.F., Lee E.A., Kuniholm A. (2019). Patient-Customized Oligonucleotide Therapy for a Rare Genetic Disease. N. Engl. J. Med..

[27] Blomqvist M., Smeland M.F., Lindgren J., Sikora P., Riise Stensland H.M.F., Asin-Cayuela J. (2019). beta-Mannosidosis caused by a novel homozygous intragenic inverted duplication in MANBA. Cold Spring Harb. Mol. Case Stud..

[28] Ashton-Prolla P., Goldim J.R., Vairo F.P., da Silveira Matte U., Sequeiros J. (2015). Genomic analysis in the clinic: Benefits and challenges for health care professionals and patients in Brazil. J. Community Genet..

[29] Pinto E.V.F., Conboy E., de Souza C.F.M., Jones A., Barnett S.S., Klee E.W., Lanpher B.C. (2018). Diagnosis of Attenuated Mucopolysaccharidosis VI: Clinical, Biochemical, and Genetic Pitfalls. Pediatrics.

[30] Bravo H., Neto E.C., Schulte J., Pereira J., Filho C.S., Bittencourt F., Sebastiao F., Bender F., de Magalhaes A.P.S., Guidobono R. (2017). Investigation of newborns with abnormal results in a newborn screening program for four lysosomal storage diseases in Brazil. Mol. Genet. Metab. Rep..

[31] Pant D.C., Dorboz I., Schluter A., Fourcade S., Launay N., Joya J., Aguilera-Albesa S., Yoldi M.E., Casasnovas C., Willis M.J. (2019). Loss of the sphingolipid desaturase DEGS1 causes hypomyelinating leukodystrophy. J. Clin. Investig..

[32] Li D., Lin Y., Huang Y., Zhang W., Jiang M., Li X., Zhao X., Sheng H., Yin X., Su X. (2018). Early prenatal diagnosis of lysosomal storage disorders by enzymatic and molecular analysis. Prenat. Diagn..

[33] Zhang J., Chen H., Kornreich R., Yu C. (2019). Prenatal Diagnosis of Tay-Sachs Disease. Methods Mol. Biol..

[34] Ou L., Przybilla M.J., Whitley C.B. (2018). SAAMP 2.0: An algorithm to predict genotype-phenotype correlation of lysosomal storage diseases. Clin. Genet..

[35] Scott H.S., Litjens T., Nelson P.V., Brooks D.A., Hopwood J.J., Morris C.P. (1992). alpha-L-iduronidase mutations (Q70X and P533R) associate with a severe Hurler phenotype. Hum. Mutat..

[36] Hein L.K., Bawden M., Muller V.J., Sillence D., Hopwood J.J., Brooks D.A. (2004). alpha-L-iduronidase premature stop codons and potential read-through in mucopolysaccharidosis type I patients. J. Mol. Biol..

[37] Nowak A., Huynh-Do U., Krayenbuehl P.A., Beuschlein F., Schiffmann R., Barbey F. (2020). Fabry disease genotype, phenotype, and migalastat amenability: Insights from a national cohort. J. Inherit. Metab. Dis..

[38] Fiehn O. (2002). Metabolomics—The link between genotypes and phenotypes. Plant Mol. Biol..

[39] Nicholson J.K., Holmes E., Kinross J.M., Darzi A.W., Takats Z., Lindon J.C. (2012). Metabolic phenotyping in clinical and surgical environments. Nature.

[40] Mussap M., Zaffanello M., Fanos V. (2018). Metabolomics: A challenge for detecting and monitoring inborn errors of metabolism. Ann. Transl. Med..

[41] Gonzalez-Dominguez A., Duran-Guerrero E., Fernandez-Recamales A., Lechuga-Sancho A.M., Sayago A., Schwarz M., Segundo C., Gonzalez-Dominguez R. (2017). An Overview on the Importance of Combining Complementary Analytical Platforms in Metabolomic Research. Curr. Top. Med. Chem..

[42] Tebani A., Abily-Donval L., Afonso C., Marret S., Bekri S. (2016). Clinical Metabolomics: The New Metabolic Window for Inborn Errors of Metabolism Investigations in the Post-Genomic Era. Int. J. Mol. Sci..

[43] Sandlers Y. (2017). The future perspective: Metabolomics in laboratory medicine for inborn errors of metabolism. Transl. Res..

[44] Wishart D.S., Feunang Y.D., Marcu A., Guo A.C., Liang K., Vazquez-Fresno R., Sajed T., Johnson D., Li C., Karu N. (2018). HMDB 4.0: The human metabolome database for 2018. Nucleic Acids Res..

[45] Mandal R., Chamot D., Wishart D.S. (2018). The role of the Human Metabolome Database in inborn errors of metabolism. J. Inherit. Metab. Dis..

[46] Auray-Blais C., Boutin M., Gagnon R., Dupont F.O., Lavoie P., Clarke J.T. (2012). Urinary globotriaosylsphingosine-related biomarkers for Fabry disease targeted by metabolomics. Anal. Chem..

[47] Auray-Blais C., Boutin M. (2012). Novel gb(3) isoforms detected in urine of fabry disease patients: A metabolomic study. Curr. Med. Chem..

[48] Manwaring V., Boutin M., Auray-Blais C. (2013). A metabolomic study to identify new globotriaosylceramide-related biomarkers in the plasma of Fabry disease patients. Anal. Chem..

[49] Dupont F.O., Gagnon R., Boutin M., Auray-Blais C. (2013). A metabolomic study reveals novel plasma lyso-Gb3 analogs as Fabry disease biomarkers. Curr. Med. Chem..

[50] Mashima R., Okuyama T., Ohira M. (2020). Biomarkers for Lysosomal Storage Disorders with an Emphasis on Mass Spectrometry. Int. J. Mol. Sci..

[51] Janeckova H., Hron K., Wojtowicz P., Hlidkova E., Baresova A., Friedecky D., Zidkova L., Hornik P., Behulova D., Prochazkova D. (2012). Targeted metabolomic analysis of plasma samples for the diagnosis of inherited metabolic disorders. J. Chromatogr. A.

[52] Jacob M., Malkawi A., Albast N., Al Bougha S., Lopata A., Dasouki M., Abdel Rahman A.M. (2018). A targeted metabolomics approach for clinical diagnosis of inborn errors of metabolism. Anal. Chim. Acta.

[53] Coene K.L.M., Kluijtmans L.A.J., van der Heeft E., Engelke U.F.H., de Boer S., Hoegen B., Kwast H.J.T., van de Vorst M., Huigen M., Keularts I. (2018). Next-generation metabolic screening: Targeted and untargeted metabolomics for the diagnosis of inborn errors of metabolism in individual patients. J. Inherit. Metab. Dis..

[54] Gertsman I., Barshop B.A. (2018). Promises and pitfalls of untargeted metabolomics. J. Inherit. Metab. Dis..

[55] Hajduk J., Matysiak J., Kokot Z.J. (2016). Challenges in biomarker discovery with MALDI-TOF MS. Clin. Chim. Acta.

[56] Hinderer C., Katz N., Louboutin J.P., Bell P., Tolar J., Orchard P.J., Lund T.C., Nayal M., Weng L., Mesaros C. (2017). Abnormal polyamine metabolism is unique to the neuropathic forms of MPS: Potential for biomarker development and insight into pathogenesis. Hum. Mol. Genet..

[57] Fu H., Meadows A.S., Ware T., Mohney R.P., McCarty D.M. (2017). Near-Complete Correction of Profound Metabolomic Impairments Corresponding to Functional Benefit in MPS IIIB Mice after IV rAAV9-hNAGLU Gene Delivery. Mol. Ther..

[58] Poswar F.O., Vairo F., Burin M., Michelin-Tirelli K., Brusius-Facchin A.C., Kubaski F., Souza C.F.M., Baldo G., Giugliani R. (2019). Lysosomal diseases: Overview on current diagnosis and treatment. Genet. Mol. Biol..

[59] Weinshilboum R. (2003). Inheritance and drug response. N. Engl. J. Med..

[60] Van Driest S.L., Shi Y., Bowton E.A., Schildcrout J.S., Peterson J.F., Pulley J., Denny J.C., Roden D.M. (2014). Clinically actionable genotypes among 10,000 patients with preemptive pharmacogenomic testing. Clin. Pharmacol. Ther..

[61] Cox T.M., Aerts J.M., Andria G., Beck M., Belmatoug N., Bembi B., Chertkoff R., Vom Dahl S., Elstein D., Erikson A. (2003). The role of the iminosugar N-butyldeoxynojirimycin (miglustat) in the management of type I (non-neuronopathic) Gaucher disease: A position statement. J. Inherit. Metab. Dis..

[62] Shapiro B.E., Pastores G.M., Gianutsos J., Luzy C., Kolodny E.H. (2009). Miglustat in late-onset Tay-Sachs disease: A 12-month, randomized, controlled clinical study with 24 months of extended treatment. Genet. Med..

[63] Peterschmitt M.J., Cox G.F., Ibrahim J., MacDougall J., Underhill L.H., Patel P., Gaemers S.J.M. (2018). A pooled analysis of adverse events in 393 adults with Gaucher disease type 1 from four clinical trials of oral eliglustat: Evaluation of frequency, timing, and duration. Blood Cells Mol. Dis..

[64] Ariceta G., Giordano V., Santos F. (2019). Effects of long-term cysteamine treatment in patients with cystinosis. Pediatr. Nephrol..

[65] Megias-Vericat J.E., Garcia-Robles A., Company-Albir M.J., Fernandez-Megia M.J., Perez-Miralles F.C., Lopez-Briz E., Casanova B., Poveda J.L. (2017). Early experience with compassionate use of 2 hydroxypropyl-beta-cyclodextrin for Niemann-Pick type C disease: Review of initial published cases. Neurol. Sci..

[66] Donida B., Raabe M., Tauffner B., de Farias M.A., Machado A.Z., Timm F., Kessler R.G., Hammerschmidt T.G., Reinhardt L.S., Brito V.B. (2020). Nanoparticles containing beta-cyclodextrin potentially useful for the treatment of Niemann-Pick C. J. Inherit. Metab. Dis..

[67] Kim Y.M., Yum M.S., Heo S.H., Kim T., Jin H.K., Bae J.S., Seo G.H., Oh A., Yoon H.M., Lim H.T. (2020). Pharmacologic properties of high-dose ambroxol in four patients with Gaucher disease and myoclonic epilepsy. J. Med. Genet..

[68] Aflaki E., Borger D.K., Moaven N., Stubblefield B.K., Rogers S.A., Patnaik S., Schoenen F.J., Westbroek W., Zheng W., Sullivan P. (2016). A New Glucocerebrosidase Chaperone Reduces alpha-Synuclein and Glycolipid Levels in iPSC-Derived Dopaminergic Neurons from Patients with Gaucher Disease and Parkinsonism. J. Neurosci..

[69] Chen Y., Jian J., Hettinghouse A., Zhao X., Setchell K.D.R., Sun Y., Liu C.J. (2018). Progranulin associates with hexosaminidase A and ameliorates GM2 ganglioside accumulation and lysosomal storage in Tay-Sachs disease. J. Mol. Med..

[70] Jian J., Hettinghouse A., Liu C.J. (2017). Progranulin acts as a shared chaperone and regulates multiple lysosomal enzymes. Genes Dis..

[71] Giugliani R., Vairo F., Kubaski F., Poswar F., Riegel M., Baldo G., Saute J.A. (2018). Neurological manifestations of lysosomal disorders and emerging therapies targeting the CNS. Lancet Child Adolesc. Health.

[72] Kishnani P., Tarnopolsky M., Roberts M., Sivakumar K., Dasouki M., Dimachkie M.M., Finanger E., Goker-Alpan O., Guter K.A., Mozaffar T. (2017). Duvoglustat HCl Increases Systemic and Tissue Exposure of Active Acid alpha-Glucosidase in Pompe Patients Co-administered with Alglucosidase alpha. Mol. Ther..

[73] Mayer F.Q., Artigalas O.A., Lagranha V.L., Baldo G., Schwartz I.V., Matte U., Giugliani R. (2013). Chloramphenicol enhances IDUA activity on fibroblasts from mucopolysaccharidosis I patients. Curr. Pharm. Biotechnol..

[74] Luddi A., Crifasi L., Capaldo A., Piomboni P., Costantino-Ceccarini E. (2016). Suppression of galactocerebrosidase premature termination codon and rescue of galactocerebrosidase activity in twitcher cells. J. Neurosci. Res..

[75] Thada V., Miller J.N., Kovacs A.D., Pearce D.A. (2016). Tissue-specific variation in nonsense mutant transcript level and drug-induced read-through efficiency in the Cln1(R151X) mouse model of INCL. J. Cell. Mol. Med..

[76] Brasell E.J., Chu L., El Kares R., Seo J.H., Loesch R., Iglesias D.M., Goodyer P. (2019). The aminoglycoside geneticin permits translational readthrough of the CTNS W138X nonsense mutation in fibroblasts from patients with nephropathic cystinosis. Pediatr. Nephrol..

[77] Baradaran-Heravi A., Balgi A.D., Zimmerman C., Choi K., Shidmoossavee F.S., Tan J.S., Bergeaud C., Krause A., Flibotte S., Shimizu Y. (2016). Novel small molecules potentiate premature termination codon readthrough by aminoglycosides. Nucleic Acids Res..

[78] Giugliani R., Dalla Corte A., Poswar F., Vancella C., Horovitz D., Riegel M., Baldo G., Vairo F. (2018). Intrathecal/Intracerebroventricular enzyme replacement therapy for the mucopolysaccharidoses: Efficacy, safety, and prospects. Exp. Opin. Orphan Drugs.

[79] Giugliani R., Giugliani L., de Oliveira Poswar F., Donis K.C., Corte A.D., Schmidt M., Boado R.J., Nestrasil I., Nguyen C., Chen S. (2018). Neurocognitive and somatic stabilization in pediatric patients with severe Mucopolysaccharidosis Type I after 52 weeks of intravenous brain-penetrating insulin receptor antibody-iduronidase fusion protein (valanafusp alpha): An open label phase 1-2 trial. Orphanet J. Rare Dis..

[80] Okuyama T., Eto Y., Sakai N., Minami K., Yamamoto T., Sonoda H., Yamaoka M., Tachibana K., Hirato T., Sato Y. (2019). Iduronate-2-Sulfatase with Anti-human Transferrin Receptor Antibody for Neuropathic Mucopolysaccharidosis II: A Phase 1/2 Trial. Mol. Ther..

[81] Schiffmann R., Goker-Alpan O., Holida M., Giraldo P., Barisoni L., Colvin R.B., Jennette C.J., Maegawa G., Boyadjiev S.A., Gonzalez D. (2019). Pegunigalsidase alfa, a novel PEGylated enzyme replacement therapy for Fabry disease, provides sustained plasma concentrations and favorable pharmacodynamics: A 1-year Phase 1/2 clinical trial. J. Inherit. Metab. Dis..

[82] Baldo G., Mayer F.Q., Martinelli B., Meyer F.S., Burin M., Meurer L., Tavares A.M., Giugliani R., Matte U. (2012). Intraperitoneal implant of recombinant encapsulated cells overexpressing alpha-L-iduronidase partially corrects visceral pathology in mucopolysaccharidosis type I mice. Cytotherapy.

[83] Xu S., Lun Y., Frascella M., Garcia A., Soska R., Nair A., Ponery A.S., Schilling A., Feng J., Tuske S. (2019). Improved efficacy of a next-generation ERT in murine Pompe disease. JCI Insight.

[84] Wasserstein M.P., Diaz G.A., Lachmann R.H., Jouvin M.H., Nandy I., Ji A.J., Puga A.C. (2018). Olipudase alfa for treatment of acid sphingomyelinase deficiency (ASMD): Safety and efficacy in adults treated for 30 months. J. Inherit Metab. Dis..

[85] Ohashi T. (2019). Gene therapy for lysosomal storage diseases and peroxisomal diseases. J. Hum. Genet..

[86] Gonzalez E B.G. (2017). Gene Therapy for Lysosomal Storage Disorders: Recent Advances and Limitations. J. Inborn Errors Metab. Screen..

[87] Corti M., Liberati C., Smith B.K., Lawson L.A., Tuna I.S., Conlon T.J., Coleman K.E., Islam S., Herzog R.W., Fuller D.D. (2017). Safety of Intradiaphragmatic Delivery of Adeno-Associated Virus-Mediated Alpha-Glucosidase (rAAV1-CMV-hGAA) Gene Therapy in Children Affected by Pompe Disease. Hum. Gene Ther. Clin. Dev..

[88] Tardieu M., Zerah M., Gougeon M.L., Ausseil J., de Bournonville S., Husson B., Zafeiriou D., Parenti G., Bourget P., Poirier B. (2017). Intracerebral gene therapy in children with mucopolysaccharidosis type IIIB syndrome: An uncontrolled phase 1/2 clinical trial. Lancet Neurol..

[89] Sessa M., Lorioli L., Fumagalli F., Acquati S., Redaelli D., Baldoli C., Canale S., Lopez I.D., Morena F., Calabria A. (2016). Lentiviral haemopoietic stem-cell gene therapy in early-onset metachromatic leukodystrophy: An ad-hoc analysis of a non-randomised, open-label, phase 1/2 trial. Lancet.

[90] Poletto E., Baldo G., Gomez-Ospina N. (2020). Genome Editing for Mucopolysaccharidoses. Int. J. Mol. Sci..

[91] Sheridan C. (2018). Sangamo’s landmark genome editing trial gets mixed reception. Nat. Biotechnol..

[92] Rinaldi C., Wood M.J.A. (2018). Antisense oligonucleotides: The next frontier for treatment of neurological disorders. Nat. Rev. Neurol..

[93] Dardis A., Buratti E. (2018). Impact, Characterization, and Rescue of Pre-mRNA Splicing Mutations in Lysosomal Storage Disorders. Genes.

[94] Rodriguez-Pascau L., Coll M.J., Vilageliu L., Grinberg D. (2009). Antisense oligonucleotide treatment for a pseudoexon-generating mutation in the NPC1 gene causing Niemann-Pick type C disease. Hum. Mutat..

[95] van der Wal E., Bergsma A.J., Pijnenburg J.M., van der Ploeg A.T., Pijnappel W. (2017). Antisense Oligonucleotides Promote Exon Inclusion and Correct the Common c.-32-13T>G GAA Splicing Variant in Pompe Disease. Mol. Ther. Nucleic Acids.

[96] Matos L., Goncalves V., Pinto E., Laranjeira F., Prata M.J., Jordan P., Desviat L.R., Perez B., Alves S. (2015). Functional analysis of splicing mutations in the IDS gene and the use of antisense oligonucleotides to exploit an alternative therapy for MPS II. Biochim. Biophys. Acta.

[97] Parenti G., Andria G., Ballabio A. (2015). Lysosomal storage diseases: From pathophysiology to therapy. Annu. Rev. Med..

[98] Macauley S.L. (2016). Combination Therapies for Lysosomal Storage Diseases: A Complex Answer to a Simple Problem. Pediatr. Endocrinol. Rev..

[99] Eisengart J.B., Jarnes J., Ahmed A., Nestrasil I., Ziegler R., Delaney K., Shapiro E., Whitley C. (2017). Long-term cognitive and somatic outcomes of enzyme replacement therapy in untransplanted Hurler syndrome. Mol. Genet. Metab. Rep..

[100] Ng M., Gupta A., Lund T., Orchard P. (2020). Impact of extended post-HSCT enzyme replacement therapy (ERT) on linear growth in mucopolysaccharidosis type IH (MPS IH). Mol. Genet. Metab..

[101] Schiffmann R., Cox T.M., Ida H., Mengel Mistry P., Crawford N., Gaemers S., Jih A., Peterschmitt M.J., Sharma J., Zhang Q. (2020). Venglustat combined with imiglucerase positively affects neurological features and brain connectivity in adults with Gaucher disease type 3. Mol. Genet. Metab..

[102] Roberts M.S., Macauley S.L., Wong A.M., Yilmas D., Hohm S., Cooper J.D., Sands M.S. (2012). Combination small molecule PPT1 mimetic and CNS-directed gene therapy as a treatment for infantile neuronal ceroid lipofuscinosis. J. Inherit. Metab. Dis..

[103] Hawkins-Salsbury J.A., Shea L., Jiang X., Hunter D.A., Guzman A.M., Reddy A.S., Qin E.Y., Li Y., Gray S.J., Ory D.S. (2015). Mechanism-based combination treatment dramatically increases therapeutic efficacy in murine globoid cell leukodystrophy. J. Neurosci..

